# What triggers healthcare-seeking behaviour when experiencing a symptom? Results from a population-based survey

**DOI:** 10.3399/bjgpopen17X100761

**Published:** 2017-04-05

**Authors:** Sandra Elnegaard, Anette Fischer Pedersen, Rikke Sand Andersen, René de-Pont Christensen, Dorte Ejg Jarbøl

**Affiliations:** 1 PhD Student, Research Unit of General Practice, Department of Public Health, University of Southern Denmark, Odense, Denmark; 2 Associate Professor, MSc, Research Unit for General Practice, Danish Research Centre for Cancer Diagnosis in Primary Care (CaP), Department of Public Health, Aarhus University, Aarhus, Denmark; 3 Associate Professor, Anthropologist, Research Unit for General Practice, Danish Research Centre for Cancer Diagnosis (CaP), Department of Public Health, Aarhus University, Aarhus, Denmark; 4 Biostatistician, Research Unit for General Practice, Department of Public Health, University of Southern Denmark, Odense, Denmark; 5 Associate Professor, Senior Researcher, GP, Research Unit of General Practice, Department of Public Health, University of Southern Denmark, Odense, Denmark

**Keywords:** signs and symptoms, general practice, behaviour and behaviour mechanisms, help-seeking behaviour, primary health care

## Abstract

**Background:**

The decision process of whether or not to contact the GP is influenced by different factors which have not all been well examined.

**Aim:**

The aim of this study was to analyse whether contact to the GP is associated with concern about the symptom, influence on daily activities and symptom burden, such as the total number of symptoms experienced by each person in a general population.

**Design & setting:**

This Danish nationwide cross-sectional study comprises a random sample of 100 000 people, representative of the adult Danish population ≥20 years.

**Method:**

Baseline data were collected in a web-based survey conducted from June to December 2012.

**Results:**

In total 49 706 (52.5%) individuals answered the questionnaire; 45 483 (91.5%) individuals experienced at least one of 44 predefined symptoms during the 4 weeks preceding the completion of the questionnaire. They reported 268 772 symptom experiences of which 58 370 symptoms (21.7%) resulted in contact with a GP. A high level of concern and influence on daily activities was associated with significantly higher odds for GP contact. A high burden of symptoms was associated with lower odds of contact with the GP.

**Conclusion:**

Approximately every fifth symptom reported by individuals from the general population leads to GP contact. Influence on daily activities, burden of symptoms, and concern about the symptom were significant factors associated with the decision of whether to contact the GP. No overall association between sex and GP contact was observed.

## How this fits in

Symptoms presented to the GP represent only an extract of the total symptom pool experienced by individuals in the general population. This study demonstrates that the decision of whether or not to contact the GP is influenced by different factors, for example, psychological, social, and behavioural factors. No overall association between sex and GP contact was observed.

## Introduction

As a health professional on the front line, the GP has contact with different kinds of people and the assessment of symptoms is a key function in primary care.

However, continuing research has illustrated that symptoms presented to the GP represent only a minority of the total symptom pool experienced by people in the general population.^1,2^ Knowledge of triggers for healthcare-seeking behaviour is key information for the GP to help them enhance the ability for early diagnosis and prompt treatment. It is important to consider that the symptoms reported to the GP will reflect a variety of interpretation of sensations, which are not always equivalent to expressions of underlying disease. Psychological factors, context, and cultural aspects also influence a patient's interpretation of bodily sensations as symptoms and these will then affect their related actions.^3^

The identification of factors associated with healthcare-seeking behaviours has been an important research area for decades and both qualitative and quantitative studies have been conducted. A review of factors associated with healthcare-seeking patterns for cancer symptoms identified a number of factors, such as demographic (age and sex), psychological (concern and fear), social (influence of family and friends), and behavioural (self-medication and watchful waiting).^4,5^ Likewise, prior studies conducted on specially selected populations, such as patients with musculoskeletal disorders or cancer, have shown associations between demographic, socioeconomic, health-related, and management factors.^6,7^

Whether a sensation is considered worrisome and a potential sign of serious disease may vary among individuals and social groups.^6^ Many aspects might contribute to this, for example, the general physical and psychological wellbeing of the person, and the cultural acceptance or indexation of symptoms.

Meanwhile, knowledge is sparse about how concern about a symptom, level of influence on daily activities and symptom burden affect the help-seeking behaviour when experiencing a symptom in an unselected general population.

Based on a survey conducted in the general population regarding symptom experiences and factors influencing the decision process of contact with the GP, the objective of this study was to analyse whether GP contact is associated with concern over the symptom, influence on daily activities, and symptom burden, such as the total number of symptoms experienced by each person in a general population.

## Method

### Study design

**Figure 1.**
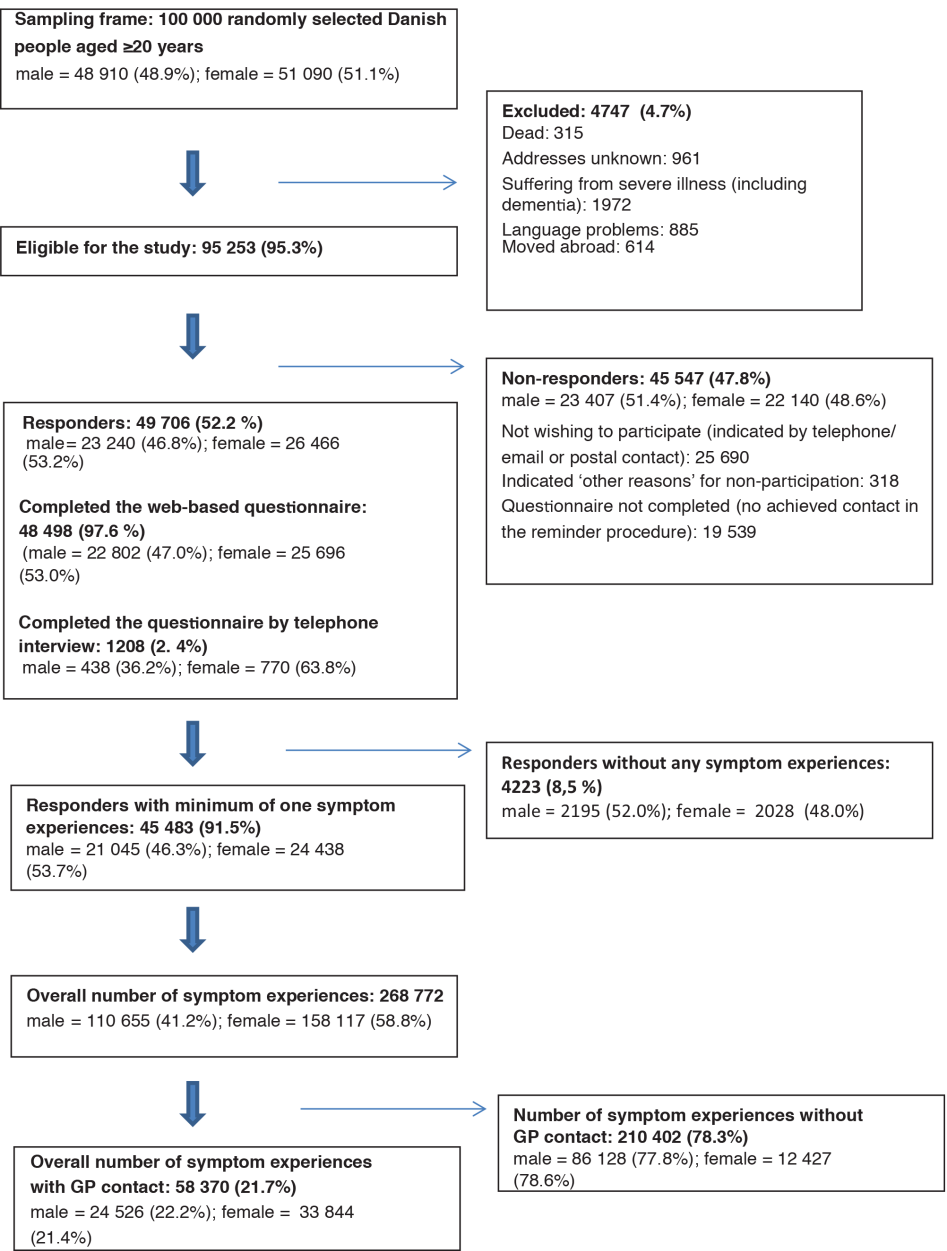
Study cohort.

This Danish nationwide cross-sectional study comprising a random sample of 100 000 people, representative of the adult Danish population aged ≥20 years Figure 1. This data is taken from the Danish Symptom Cohort.^8,9^ Baseline data presented in this study was collected in a web-based survey. The data collection was conducted from June to December 2012.

### Subjects and sampling

All Danish citizens are registered with a unique personal identification number in the Danish Civil Registration System (CRS), which contains information on any resident’s date of birth, sex, ethnicity, and migration.^10^ The sample for this study was randomly selected using the CRS and participants were invited to take part in the survey. Each individual received a postal letter explaining the purpose of the study. In the letter, a unique 12-digit login to a secure webpage was included. This provided access to a comprehensive web-based questionnaire. In order to prevent the exclusion of people with no access to a computer, tablet, or smartphone, the participants were offered the opportunity to respond to the survey via telephone interview.

### Questionnaire

**Figure 2.**
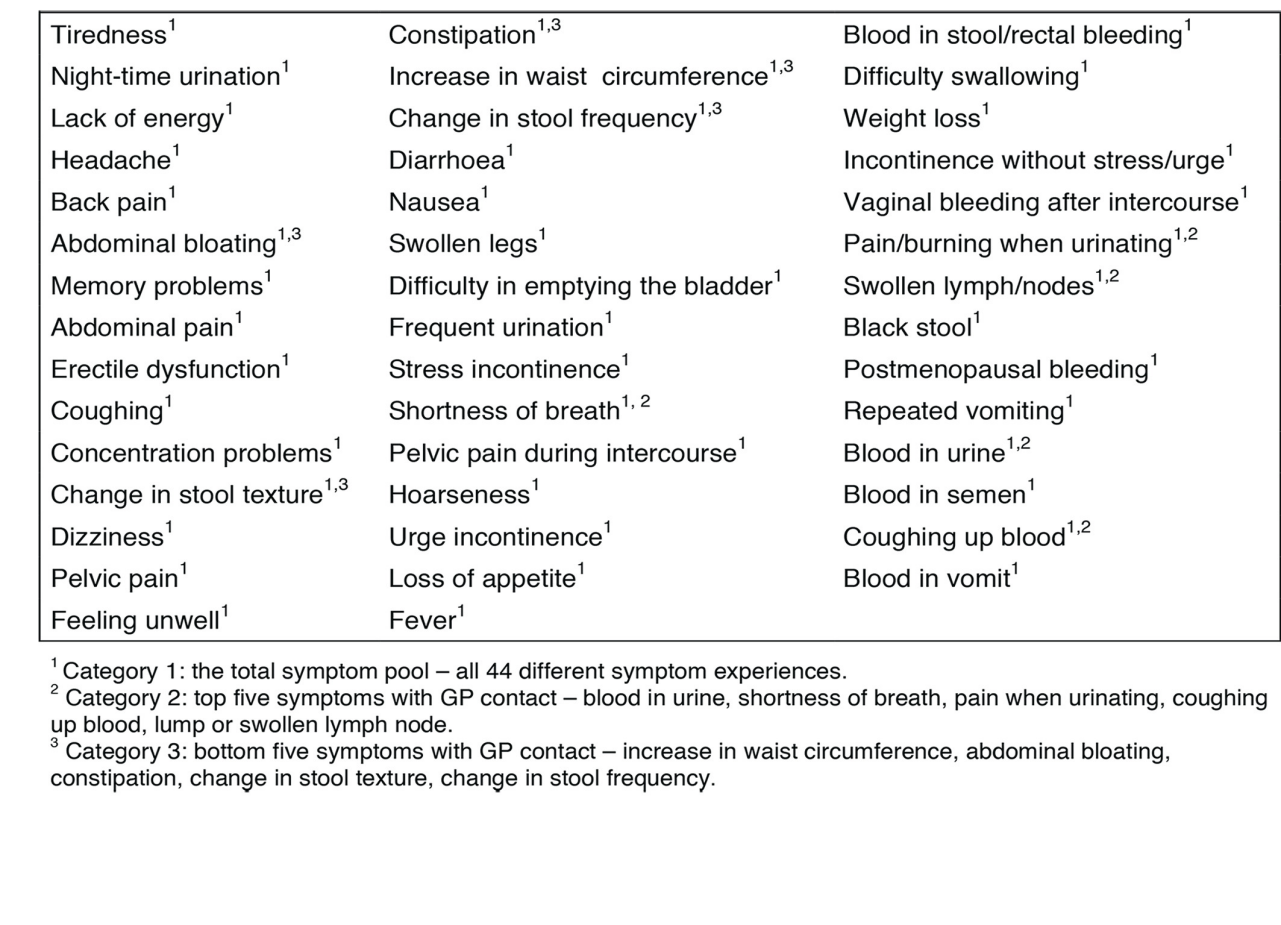
The 44 predefined symptoms from the questionnaire.

To explore the prevalence of different symptom experiences and subsequent healthcare-seeking behaviours, a comprehensive questionnaire including 44 different predefined symptoms was constructed (Figure 2). The questionnaire was pilot- and field-tested and adjusted accordingly. The methodological framework for developing the questionnaire is described in the data analysis section.^8^

Items regarding each specific symptom were phrased: ‘Have you experienced the following bodily sensation, symptom, or discomfort within the past 4 weeks?’ With regard to GP contact, the question for each selected symptom was: ‘Have you contacted your GP with the following symptom or discomfort, in person, by phone or by email?’

Items concerning the extent of the symptom(s) or discomfort(s) interfered with the usual daily activities and to what extent the responder was concerned about the symtom(s), were also included. Furthermore, a question adressing general concern about the current health, was phrased as ‘To what extent are you concerned about your current health?’.

### Data analysis

The dataset used in the analyses was constructed by merging all reported symptoms experienced by the responders with each individual symptom experience. This was then used as a study case. For each symptom experience, the outcome variable was whether or not the GP had been contacted about the symptom. To assess the appropriateness of merging the 44 different symptom experiences, the researchers conducted sensitivity analyses considering each of the 44 symptom experiences separately.

Covariates considered in the uni-, bi-, and multivariable statistical analyses were; influence on daily activities, concern about the symptom, and symptom burden. Influence on daily activities, concern about the symptom and general concern about the current health were categorised on a 5-point scale: ‘none at all’, ‘slight’, ‘moderate’, ‘quite a bit’, and ‘extreme’.

The number of symptoms experienced by each individual was used as a proxy for the symptom burden. Thus, for each symptom experience reported by the same individual, the symptom burden was constant. Furthermore, the symptom burden was categorised into five groups: 1–2, 3–5, 6–10, 11–15 and ≥16 symptom experiences. The a priori hypothesis regarding ‘burden of symptom’ was that people experiencing many symptoms, regardless of the type of symptoms, had a higher utilisation of the GP. This was notably the case for women experiencing many symptoms. This was also the reason for the analysis of the population based on the 44 different symptoms experienced independently of diagnosis.

The effect of age was initially explored by dividing individuals into 5-year age groups, but as results indicated estimates to be homogeneous within 20-year categories, the authors reported results only in the following 20-year age groups: 20–39, 40–59, 60–79, and ≥80 years.

Based on the previous work^10^, three categories of symptoms were defined. Category 1 embraces all 44 symptom experiences and comprises the total symptom pool. Category 2 encompasses the five symptoms with the highest proportion of GP contact. Category 3 includes the five symptoms with the lowest proportion of GP contact (Figure 2). The two latter categories were chosen with the purpose of exploring the robustness of results in different groups of symptom experiences, particularly with regard to severity and general symptoms experienced and reported in general practice.

Prevalence estimates of self-reported symptom experiences and the proportions of subsequent contact with the GP were calculated. For each covariate, differences between the distribution of symptom experiences with or without GP contact were tested using a χ^2^ test.

Logistic regression models were used to analyse associations between GP contact and the covariates. Robust cluster estimation was used to account for dependency between symptoms experienced by the same individual. Adjustments were made for possible confounders: sex, general concern, and symptom burden. To evaluate collinearity between covariates, correlation coefficients were calculated with Spearman’s rank correlation. Logistic regression models were used to test for interaction between age, sex, and each covariate with regard to GP contact with a symptom experience. Owing to interactions, the analyses were stratified with respect to age. No formal methods were employed to handle multiple testing. Odds ratios (ORs) are presented with 95% confidence intervals. A *P*-value <0.05 was considered statistically significant. All data analyses were conducted using STATA statistical software 13.1.

## Results

Of the 100 000 randomly selected subjects, 4 474 (4.7%) were not eligible. Of the 95 253 (95.3%) eligible subjects, 49 706 individuals completed the questionnaire, yielding a response rate of 52.2% (Figure 1). The responders were fairly representative of the study sample according to ethnicity, socioeconomic, and demographic variables, which are described in details elsewhere.^10^

Some 45 483 (91.5%) individuals experienced at least one of the predefined symptoms during the 4 weeks preceding the completion of the questionnaire. They reported a total of 268 772 symptom experiences of which 58 370 symptoms (21.7%) resulted in contact to a GP (Figure 1).

Prevalence estimates of self-reported symptom experiences during the preceding 4 weeks and the proportions of GP contact apportioned between the different covariates are listed in Table 1. Individuals with the highest prevalence of symptom experiences were female (mean number of symptoms 6.0), individuals in the young age group of 20–39 years (mean number of symptoms 6.5), and in the oldest age group of ≥80 years (mean number of symptoms 5.6). Proportions of symptoms with GP contact were reported almost equally by males 24 526 (22.2%) and female 33 844 (21.4%), and to greater extent reported by individuals in the age group of ≥80 years *n* = 2 849 (36.0%). The proportion of GP contact with a symptom was higher for symptoms reported as extremely concerning (58.3%), with an extreme level of influence on daily activities (49.7%) and by individuals with a symptom burden of ≥16 symptoms (30.9%). The proportion of symptoms that led to GP contact differed significantly with respect to all covariates (Table 1).

**Table 1.** Characteristics of study sample with regard to symptoms and GP contact

	Study sample		Number of symptoms			Number of symptoms with GP contact		
	*n*	%	*n*	%	Mean (SD)	*n*	%	*P*-value^a^
**Study sample**								
Overall	49 706	100.0	268 772	100.0	5.4 (4.39)	58 370	21.7	
**Sex**								<0.001
Male	23 240	46.8	110 655	41.2	4.8 (3.94)	24 526	22.2	
Female	26 466	53.2	158 117	58.8	6.0 (4.67)	33 844	21.4	
**Age, years**								<0.001
20–39	12 251	24.6	80 026	29.8	6.5 (4.69)	12 145	15.2	
40–59	20 305	40.9	109 196	40.6	5.4 (4.32)	21 822	20.0	
60–79	15 748	31.7	71 639	26.7	4.6 (4.01)	21 554	30.1	
≥80	1 402	2.8	7 911	2.9	5.6 (4.43)	2 849	36.0	
**Symptom burden** (Total number of symptoms experienced)								<0.001
0	4 223	8.5						
1–2	10 524	21.2	15 951	5.9		2 582	16.2	
3–5	14 878	29.9	58 577	21.8		10 486	17.9	
6–10	13 589	27.3	103 596	38.5		21 264	20.5	
11–15	4 930	9.9	61 772	23, 0		15 126	24.5	
≥16	1 562	3.1	28 876	10.7		8 912	30.9	
**Influence on daily activities**								<0.001
None at all			43 989	16.5		4 287	9.75	
Slight			92 3304	34.7		13 112	14.2	
Moderate			64 642	24.3		15 186	23.5	
Quite a bit			43 905	16.5		15 118	34.4	
Extreme			21 452	8.1		10 667	49.7	
**Symptom concern**								<0.001
None at all			100 615	37.9		9 478	9.4	
Slight			80 504	30.3		15 562	19.3	
Moderate			44 538	16.8		13 974	31.3	
Quite a bit			26 427	10.0		11 640	44.1	
Extreme			13 272	5.0		7 743	58.3	

^a^Percentages might not fully match with total numbers due to missing information. Missing information does not exceed 1%. Tested for difference between groups with χ^2^ test. SD = standard deviation.

When dividing the 44 different symptoms into three categories with regard to proportion of GP contact, the lowest and highest proportions, influence on daily activities and concern about the symptom, were highly associated with GP contact in all three categories. Category II encompassing: blood in urine, shortness of breath, pain when urinating, coughing up blood, and lump or swollen lymph node, indicated decreasing ORs for GP contact with increasing symptom burden (Table 2).

**Table 2.** Odds ratios for GP contact with a symptom stratified in three different symptom categories

	OR for GP contact with a symptom									
	Category 1: All symptoms			Category 2: Top five symptoms concerning likelihood of GP contact			Category 3: Bottom five symptoms concerning likelihood of GP contact			
	OR crude	AOR	95% CI	OR crude	AOR	95% CI	OR crude	AOR	95% CI	
**Sex^a^**										
Male	1	1	–	1	1	–	1	1	–	
Female	0.96	1.02	0.97 to 1.06	1.00	1.18	1.05 to 1.33	0.98	0.98	0.89 to 1.08	
**Age, years^a^**										
20–39	1	1	–	1	1	–	1	1	–	
40–59	1.39	1.23	1.16 to 1.30	1.42	1.28	1.11 to 1.49	1.27	1.13	1.00 to 1.28	
60–79	2.44	2.21	2.09 to 2.34	2.77	2.46	2.10 to 2.87	2.42	2.07	1.83 to 2.34	
≥80	3.38	3.15	2.78 to 3.56	3.65	3.51	2.54 to 4.86	4.45	3.86	2.97 to 5.02	
**Influence on daily activities^b^**										
None at all, reference	1	1	–	1	1	–	1	1	–	
Slight	1.53	1.49	1.42 to 1.57	1.11	1.07	0.89 to 1.29	1.85	1.66	1.46 to 1.89	
Moderate	2.86	2.65	2.51 to 2.79	1.78	1.72	1.42 to 2.08	3.73	3.11	2.71 to 3.55	
Quite a bit	4.89	4.35	4.12 to 4.60	2.89	2.72	2.22 to 3.34	6.49	5.06	4.37 to 5.87	
Extreme	9.33	7.62	7.12 to 8.15	4.84	4.25	3.31 to 5.46	13.34	9.67	8.01 to 11.69	
**Symptom concern^b^**										
None at all, reference	1	1	–	1	1	–	1	1	–	
Slight	2.31	2.12	2.03 to 2.22	1.36	1.42	1.20 to 1.70	2.53	2.24	2.00 to 2.50	
Moderate	4.40	3.82	3.63 to 4.02	2.03	2.12	1.76 to 2.55	5.35	4.40	3.88 to 5.00	
Quite a bit	7.59	6.40	6.04 to 6.79	3.22	3.53	2.89 to 4.32	10.03	7.89	6.78 to 9.20	
Extreme	13.53	10.74	9.94 to 11.61	5.40	5.78	4.54 to 7.37	15.78	12.01	9.71 to 14.85	
**Symptom burden (total number of symptoms experienced)^a^**										
1–2, reference	1	1	–	1	1	–	1	1	–	
3–5	1.13	0.89	0.86 to 0.94	0.82	0.75	0.54 to 1.05	1.24	1.10	0.85 to 1.43	
6–10	1.34	0.86	0.81 to 0.92	0.67	0.55	0.40 to 0.76	1.67	1.20	0.93 to 1.55	
11–15	1.69	0.85	0.79 to 0.92	0.59	0.43	0.31 to 0.61	2.45	1.32	1.01 to 1.72	
≥16	2.36	0.97	0.87 to 1.08	0.60	0.45	0.31 to 0.64	4.00	1.58	1.19 to 2.10	

**Category 1 = **total symptom pool: all 44 different symptom experiences. **Category 2 = t**op five symptoms with GP contact: blood in urine, shortness of breath, pain when urinating, coughing up blood, and lump or swollen lymph node. **Category 3 = **bottom five symptoms with GP contact: increase in waist circumference, abdominal bloating, constipation, change in stool texture, and change in stool frequency. AOR = adjusted odds ratio. OR = odds ratio. ^a^Adjusted for general concern, concern about the symptom, influence on daily activities, age and sex.^ b^Adjusted for general concern, symptom burden, age and sex.

ORs for GP contact with a symptom according to each of the three covariates, stratified on age are shown in Table 3. Similarly to the overall findings, the results were rather consistent over age groups. ORs for GP contact with a self-reported symptom increased almost consistently with the concern about the symptom in all age groups. The same applied to GP contact with a symptom reported as influencing the daily activities. However, higher ORs for GP contact were seen among the younger individuals reporting extreme concerns for the symptoms and extreme influence on daily activities (Table 3).

**Table 3.** OR for GP contact with a symptom, stratified with respect to age groups

	Age groups, years										
	All			20–39		40–59		60–79		≥80	
	OR crude	AOR^a^	95% CI	AOR^b^	95% CI	AOR^b^	95% CI	AOR^b^	95% CI	AOR^b^	95% CI
**Influence on daily activities**											
None at all, reference	1	1	–	1	–	1	–	1	–	1	–
Slight	1.53	1.49	1.42 to 1.57	1.77	1.59 to 1.97	1.48	1.37 to 1.61	1.39	1.28 to 1.50	1.74	1.40 to 2.16
Moderate	2.86	2.65	2.51 to 2.79	3.05	2.73 to 3.41	2.85	2.61 to 3.11	2.38	2.19 to 2.59	2.17	1.70 to 2.77
Quite a bit	4.89	4.35	4.12 to 4.60	5.18	4.60 to 5.82	4.52	4.12 to 4.97	3.97	3.61 to 4.35	3.50	2.71 to 4.52
Extreme	9.33	7.62	7.12 to 8.15	9.57	8.35 to 10.98	7.93	7.09 to 8.86	6.51	5.79 to7.30	6.11	4.56 to 8.20
**Symptom concern**											
None at all, reference	1	1	–	1	–	1	–	1	–	1	–
Slight	2.31	2.12	2.03 to 2.22	2.52	2.29 to 2.77	2.36	2.18 to 2.55	1.75	1.62 to 1.88	1.36	1.10 to 1.70
Moderate	4.40	3.82	3.63 to 4.02	4.62	4.16 to 5.13	4.48	4.11 to 4.88	3.04	2.79 to 3.31	1.89	1.48 to 2.40
Quite a bit	7.59	6.40	6.04 to 6.79	7.78	6.90 to 8.77	7.50	6.80 to 8.27	4.98	4.52 to 5.48	3.10	2.33 to 4.11
Extreme	13.53	10.74	9.94 to 11.61	13.17	11.23 to 15.45	12.03	10.59 to 13.67	8.76	7.70 to 9.97	5.00	3.50 to 7.15
**Symptom burden (total number of symptoms experienced)**											
1–2, reference	1	1	–	1	–	1	–	1	–	1	–
3–5	1.13	1.08	1.02 to 1.15	0.86	0.73 to 1.02	0.85	0.77 to 0.94	0.96	0.88 to 1.05	1.01	0.73 to 1.39
6–10	1.34	1.22	1.15 to 1.29	0.91	0.77 to 1.07	0.77	0.69 to 0.85	0.96	0.97 to 1.06	1.10	0.79 to 1.51
11–15	1.69	1.38	1.28 to 1.48	0.88	0.74 to 1.06	0.78	0.69 to 0.88	0.92	0.81 to 1.04	1.20	0.80 to 1.79
≥16	2.36	1.76	1.60 to 1.95	0.95	0.77 to 1.18	0.90	0.76 to 1.07	1.02	0.83 to 1.25	1.24	0.73 to 2.10

^a^Adjusted for general concern, symptom burden, age, and sex. ^b^Adjusted for general concern, symptom burden and sex.

The sensitivity analysis considering each of the 44 symptom experiences separately showed similar results as the merged group of symptoms for the majority of symptoms. Only a few symptoms resulted in slightly higher ORs for GP contact, however, with wide confidence intervals.

## Discussion

### Summary

The decision process of whether to contact the GP with a symptom was influenced by level of symptom concern, level of influence on daily activities and symptom burden. The level of influence on daily activities and the level of concern were more strongly associated with GP contact in the younger age groups. Symptom burden was negatively associated with GP contact when adjusted for the other covariates, such as general concern, age, and sex. This was not the case for the category of symptoms with the lowest proportion of GP contact where the symptom burden increased odds for GP contact.

### Strengths and limitations

This study was a large cross-sectional nationwide population-based study, including 100 000 people randomly selected from the Danish CRS register, representative of the adult population aged ≥20 years. To the authors' knowledge, such a large-scale nationwide population-based study, investigating a wide range of self-reported symptom experiences and subsequent GP contacts covering 44 different predefined symptoms, has not previously been conducted.

The response rate of 52.2% was comparable or even higher compared to previous surveys measuring symptom prevalence in the general population.^11,12^ Although a preponderance of the responders were female, and slightly older than the non-responders, they were fairly representative of the general Danish population. Differences between respondersand non-responders regarding other parameters, such as risk of over or underestimating the proportion of GP contacts, cannot be eliminated. For more details, see Elnegaard and colleagues.^9^

To avoid a possible selection bias due to the web-based design of the questionnaire, participants without access to a computer, smartphone, or tablet were offered the possibility of conducting the survey via telephone interview. All the data extracted from the questionnaire was self-reported and assumed to reflect the individuals’ authentic experiences of symptoms. A qualitative approach might reflect an even more authentic picture of the symptom experience, but this method also has limitations. The participants were asked to recall symptom experiences within the preceding 4 weeks and whether they, at any time, had contacted a GP with these symptoms. Recall bias cannot be completely eliminated in questionnaire studies.^13^ Some participants may misplace older symptom experiences in the specified timeframe due to the severity of symptoms, or because they had previously contacted a GP about them.^14^ Others may have forgotten about a symptom experience or a GP contact, because the symptom turned out to be nothing to worry about or simply due to loss of memory.^15^ The recall period was chosen to ensure getting enough symptom experiences to obtain statically precise estimates, even for rare symptoms, while still assuming that individuals could recall symptoms and GP contact fairly accurately within this timespan.^16,17^

A few gender-specific symptoms were included in the questionnaire, but for the majority of the 44 different symptoms, both men and women were asked the same questions. This contrasts with studies with only gender-specific symptoms included or special groups of patients invited to participate in the study.^7^

The sensitivity analyses conducted prior to the merging of the 44 predefined symptoms indicated a minimum variation among the symptom experiences and GP contacts with regard to concerns and influence on daily activities. Based on these results, merging the different symptom experiences was reasonable.

### Comparison with existing literature

Triggers in the decision process whether or not to contact the GP are reported and analysed in previous research,^6,7,18,19^ but few studies have estimated symptom burden, symptom concern, and influence on daily activities in an adult general population aged 20–101 years. In a systematic review regarding delayed presentation of symptomatic cancer and a recent primary care based survey, triggers of help-seeking behaviour were identified as the worsening of symptoms, new additional symptoms affecting daily life and the influence of family and friends.^6,20,21^ Whitaker and colleagues^19^ found similar results as in the present study, but in a primary care sample of individuals aged >50 years reporting alarm symptoms. However, ORs were substantially lower in the Whitaker study.^19^ Differences in age groups, timeframes and the type of symptoms might explain some of these differences.

A growing body of gender-specific studies expresses differing trends towards gender as a determining factor in the decision to contact the GP with a symptom.^22–25^ Few studies have examined consultation rates among men and women known to have comparable morbidity, and found that despite higher daily rates of symptoms for women, a great commonality in how men and women react to common bothersome symptoms was found.^18^ In the present study, no overall difference in GP contact was found between symptoms experienced by men and women. The same was recognised in the study by Elliot and colleagues,^26^ who in a population of individuals aged <60 years found that men and women contacted a GP with the same frequency when experiencing a symptom.

Overall, the likelihood of GP contact with a symptom experience increased significantly with age, but level of influence on daily activities and level of concern were more strongly associated with GP contact in the younger age groups. These findings are contrary to McAteer and colleagues.^16^ A reason for this could be the fact that the participants in McAteer’s study ranged from 18–60 years of age, whereas in the present study, researchers also included older age groups.

When categorising the reported symptoms into three categories with regard to the proportion of GP contact, only a minor divergence was seen in ORs. A stronger association between concern, influence on daily activities, and GP contact was seen in the category representing the symptoms with the lowest proportion of GP contact. This category of symptoms represents the most commonly experienced symptoms in the population, which might be part of the explanation meaning that the experience of a common symptom involves a higher level of concern and influence on daily activities before the GP is contacted.

### Implications for research and practice

In conclusion, this study emphasises coherences in the decision process of whether to contact a GP, for example symptom concern and influence on daily activities are large triggers of healthcare-seeking behaviour, overall independent of gender and age. Despite the high ORs as triggers of GP contact, only half of the symptoms reported as extremely concerning or having extreme influence on daily activities resulted in contact with a GP. The study confirms that symptoms presented to the GP only represent an extract of the total symptom pool. Whether this is the ‘correct’ healthcare-seeking behaviour is not easily discernible, but the findings do indicate that people seek medical advice when they worry that a sensation may be a sign of disease and when daily activities are troubled by the symptom. From a population perspective, this seems reasonable.

From a general practice perspective, it is important to facilitate the increasingly substantial interface between people's management of their own health and the care that is provided in collaboration with, or by, healthcare professionals.
